# Challenges in substance use treatment as perceived by professionals and Arabic-speaking refugees in Germany

**DOI:** 10.1186/s13011-023-00576-5

**Published:** 2023-11-17

**Authors:** Ebtesam A. Saleh, Felix Klapprott, Andreas Heinz, Ulrike Kluge

**Affiliations:** 1 Department of Psychiatry and Psychotherapy, Charité-Universitätsmedizin Berlin, Campus Charité Mitte (CCM), Charitéplatz 1, 10117 Berlin, Germany; 2 Department of Pharmacology, Faculty of Pharmacy, University of Aden, Aden, Yemen; 3 Berlin Institute for Integration and Migration Research (BIM), Humboldt-Universität zu Berlin, Berlin, Germany

**Keywords:** Substance use, Refugees, Interviews, Qualitative, Treatment

## Abstract

**Background:**

Substance use (SU) and substance use disorders (SUDs) have been recently documented among forcibly displaced populations as a coping mechanism to migration and postmigration stressors. Although the literature exploring substance use among refugees has grown recently, little is known about SU among Arabic-speaking refugees and, more specifically, on the challenges and experiences in regards to SU treatment. This study investigates this topic from the perspectives of Arabic-speaking refugees and professionals in Germany.

**Methods:**

**Design and participants** To expand our knowledge on this topic, a qualitative approach was employed by conducting in-depth and semi-structured interviews among 26 participants (13 refugees and 13 professionals) in Germany during 2020–2021. Purposive sampling was used to recruit Arabic-speaking refugees in two rehabilitation centers in Berlin.

**Data and analysis** Interviews were conducted with 26 participants of which 13 were refugees and 13 professionals. Refugees were interviewed individually in the rehabilitation centers, they ranged from 21 to 52 years of age, and their average time in Germany was 6.3 years. An open-ended survey was conducted among the professionals via the SoSci-survey platform, and they ranged from 22 to 66 years of age, with an average of 5 to 9 years of work experience. Data were analyzed using thematic analysis.

**Results:**

Three themes resulted from the thematic analysis: (1) The treatment is facilitated by institutional and emotional support; (2) The affected refugees struggle with complex contextual barriers to access SUD treatment; and (3) Individual and community preventive strategies are needed.

**Conclusions:**

This study provides insight into the support and challenges of accessing effective SU treatment and prevention among Arabic-speaking refugees in Germany. Collaborative efforts by the community, professionals, and policymakers are needed to facilitate access to effective treatment and implement culturally and linguistically sensitive approaches for the treatment and prevention of SU among refugees.

**Supplementary Information:**

The online version contains supplementary material available at 10.1186/s13011-023-00576-5.

## Introduction

The Federal Office for Migration and Refugees has documented that Arabic-speaking refugees constitute Germany’s largest refugee group [1]. Around 2 million Arabic-speaking refugees fled to Germany, came from war-torn countries (e.g., Syria, Libya, Yemen, Somalia, and Iraq), and struggled with war and conflict-related violence, family member loss, murder, torture, and poverty [2, 3]. In addition to migration flight hardships, they are challenged with post-migration stressors (e.g., legal status, housing conditions, language barriers, and unemployment), making them vulnerable to substance use (SU) and substance use disorders (SUDs) as a coping mechanism for psychological distress, such as post-traumatic stress disorder (PTSD) and depression [4, 5]. That is, they have experienced traumatic events that can contribute to substance use [6]. Furthermore, the attitude of Arabic-speaking refugees toward substance use and treatment is influenced by several cultural factors (e.g., religion and social norms) impacting their attitudes toward substance use and treatment. According to Lindert et al. [7], Syrian male refugees reported different societal norms and rules in Germany compared to their home country. However, our understanding is still limited regarding norms and other substances that might be socially accepted in other Arabic countries.

SUDs are defined as the use of alcohol, pharmaceuticals, or street drugs^1^ at a clinically significant level, as indicated by key symptoms, such as tolerance development, withdrawal, craving, and loss of control, which can be interpreted as impairments in key functions relevant to human life and survival, as well as individual harm caused by this consumption [8]. A high prevalence of SU and psychoactive substance consumption among the German population has been reported and is considered a significant burden to society [9]. Literature documented a higher rate of mental health problems among Arabic-speaking refugees in Germany due to post-migration stressors [5, 10], and sleep disturbance has been documented among Arabic-speaking refugees as an indicator of mental stressors [11]. Despite the need for psychological treatment for the distressed population, evidence has shown that only a minority of refugees in Europe effectively utilize mental health care and SUD treatment services [12]. Complex challenges emerge when connecting refugees to treatment services [6]. Recent studies indicated that institutional barriers in Germany hinder refugees’ access to effective prevention and treatment of SUDs [13, 14]. In addition, data on the prevalence and treatment outcomes of substance use among Arabic-speaking refugees in Germany are still scarce.

Several relevant studies employed explanatory models, where the quantitative findings identified variations in prevalence based on ethnic and age groups, study settings, and types of substances used [15, 16]. The investigation of SU as a cross-culturally sensitive topic is influenced by psychological, social, and cultural factors, including legalization and socially accepted substances among different communities [6, 17]. The standardized questions and scales of quantitative instruments may be prone to misinterpretation when employed among diverse groups owing to sociocultural variations [6]. For instance, Iraqi refugees in the United States reported difficulties in understanding the diversity in drug policies and availability of substances and had misconceptions and confusion about relevant substance-use terminologies such as heavy drinking, alcoholism, substance misuse, abuse vs. overuse, recreational use, and so on [18].

It is crucial to understand how refugees perceive the treatment for SUDs in the host country. Although insomnia is one of the most common problems during substance use treatment, it has rarely been reported among refugees receiving treatment for substance use. Insomnia can negatively impact the treatment, interfere with the ability to engage in therapy, and contribute to increased cravings and risk of relapse [19]. Understanding the factors contributing to insomnia among refugees undergoing substance use treatment is significant for effective treatment planning.

In qualitative studies, poor knowledge about the SUD treatment process has been reported among ethnic refugee groups (e.g., Afghan, African, Karen, and Iraqi) in the United States and Australia [6]. Contextual variations require an in-depth exploratory approach that provides a better understanding of the interplay of micro- and macro-dimensions of challenges in accessing treatment for SUD, as perceived by different cultural groups, specifically in countries that host multicultural groups [20]. Although investigating the perspectives of target groups on mental health has been recommended by the UNHCR [21], in-depth qualitative findings on the experiences and perceptions of SUD treatment among Arabic-speaking refugees and professionals remain scarce [6]. Given the complex nature and sensitivity of this topic, Arabic cultural and linguistic variations are considered the main challenges in conducting similar studies on host communities [22, 23].

To facilitate effective and acceptable support for refugees, it is crucial to understand how a specific ethnic refugee group perceives culturally and linguistically sensitive issues such as SU and treatment [24, 25]. Although much research has focused on SU among refugees, a much smaller body of literature has addressed substance use among Arabic-speaking refugees in host communities [6], specifically in treatment settings, from the perspectives of refugees and professionals. Accordingly, this study aimed to advance knowledge about SUD treatment experience in terms of the challenges, facilitators, and potential preventive strategies as perceived by affected refugees and professionals in Germany. In addition, insomnia in this population might be influenced by various factors, including substance withdrawal and psychological distress [26], so identifying these factors can help inform the development of targeted interventions to improve sleep and overall treatment outcomes.

Hereby, this study focuses on the following questions: “How do refugees and professionals perceive the facilitators and challenges of SU treatment in Germany?”, “How do refugees perceive their sleep quality during treatment as one of the treatment outcomes?”, and “How do they perceive potential preventive strategies regarding substance use?”

## Methods

Integrating different perspectives on this problem will help provide a better understanding of the research question(s); therefore, we employed in-depth interviews among refugees and an open-ended survey among professionals to contextualize this issue from the perspectives of both groups. Data from both populations will strengthen the results by providing a triangulation of data sources and help develop a comprehensive understanding of the phenomenon [27].

### Participant recruitment

Arabic-speaking refugees receiving treatment for SU in Berlin were recruited through purposive sampling. The inclusion criteria for potential participants were adult Arabic-speaking refugees, legal status as refugees, and receiving treatment for substance use in the late stages of treatment [28]. Furthermore, professionals’ perspectives were included to provide an in-depth understanding of the situation from different perspectives, as previously carried out to describe SU in smaller samples [29]. The inclusion criteria regarding the professionals were that they were residents in Germany and social and health workers who have working experience with Arabic-speaking refugees.

#### Refugee sample

After several visits and contact with four counseling and rehabilitation centers, participants were recruited from two rehabilitation centers in Berlin: residential and non-residential services.^2^ The administrative staff facilitated recruitment by preparing an anonymized list of candidates (10 from the non-residential center and 9 from the residential center). The interviewer introduced the project to the refugees who use substances (17 men and 2 women) during their regular clinic visits. Five candidates declined. Interviews were scheduled with those who agreed to participate (13 men and 1 woman). The only woman in our sample relapsed with a high dose of cocaine and was referred to the emergency room. After reading the study information and data protection, 13 men approved their participation and signed the consent form (in the Arabic language). Interviews were conducted inside the rehabilitation centers as the participants are familiar with the clinic environment. A psychosocial support was available in cases of re-traumatization or emotional distress triggered during the interview. This support was provided by a specialist working inside the rehabilitation centers.

#### Professionals sample

The professional sample consisted of health and social service providers working in the same rehabilitation center (NOKAT and GUIDANCE) and other relevant treatment and social services in Berlin. An invitation email with the study information was sent to the administration staff of five rehabilitation and counseling centers in Berlin for refugees. We sent the survey link to the professionals who agreed to participate. The study information and consent form were written in three languages (Arabic, English, and German) via the SoSci survey platform.

### Data collection

Two types of data were collected. First, qualitative interviews were done with refugees and professionals. A total of 26 interviews were conducted in 2020 and 2021. The interviews with refugees were conducted individually with each participant, audio-recorded, translated, and transcribed verbatim. The participants’ primary data were documented based on their profiles in the rehabilitation services and the introduction questions of the interview. The interview questions focused on refugees’ experiences with SU treatment, facilitators, challenges, and preventive strategies. Additionally, we asked professional participants to provide written responses to 16 open-ended questions regarding their experiences on substance use treatment among Arabic speaking refugees. The questions were asked via the online open-ended survey ‘SoSci-Survey’ (Supplementary File 1). Collecting data from two sources (professionals and refugees) aimed to enhance confidence in the findings, in line with the idea of “data triangulation” [27, 30]. To include multiple perspectives on the data and deepen reflexive engagement with the responses, more than one researcher was involved in interpreting findings through “investigator triangulation” [30].

Also, there was a self-reported instrument, the Insomnia Severity Index (ISI), which measures the participant’s perception of insomnia severity, distress, and daytime impairment. Suleiman and Yates [31] validated and translated ISI into Arabic. As a treatment outcome measurement, the ISI helps in providing measurable data that is complementary to the qualitative data regarding the treatment challenges and outcomes. It has been used in SU research [32, 33] and its significant value includes assessment questions about sleep quality during qualitative interviews with patients undergoing treatment for SU [32]. The ISI evaluates seven dimensions: severity of sleep onset, sleep maintenance, early morning awakening problems, sleep dissatisfaction, interference of sleep difficulties with daytime functioning, and noticeability of sleep difficulties (Supplementary File 2). The ISI was employed previously among Arabic-speaking population [32, 34] and for our study, it provides further measurable data on the sleeping quality as one of the treatment outcomes and helped us initiate conversations with the participants about their health, which promoted communication and mutual trust [35]. The ISI is seven-item scale measuring insomnia symptoms with scores ranging from 0 to 28. A 5-point Likert scale is used to rate each item (e.g., 0 = no problem to 4 = very severe problem). The total score is interpreted as follows: absence of insomnia (0–7), sub-threshold insomnia (8–14), moderate insomnia (15–21), and severe insomnia (22–28). The internal consistency of ISI in this study was excellent (Cronbach’s alpha 0.92).

### Data analysis

**Fig. 1:**
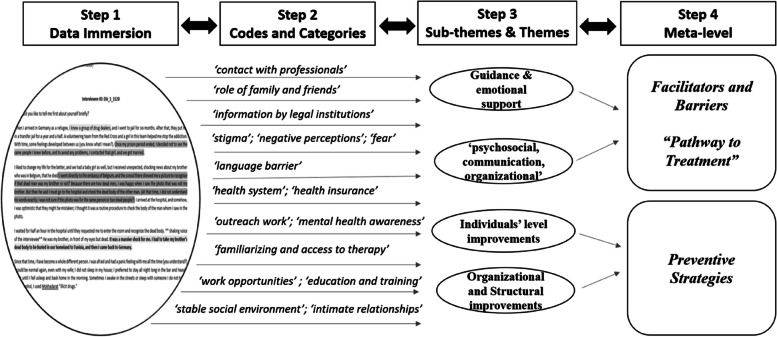
A descriptive chart of the thematic analysis conducted for 26 interviews in this study

**Fig. 2:**
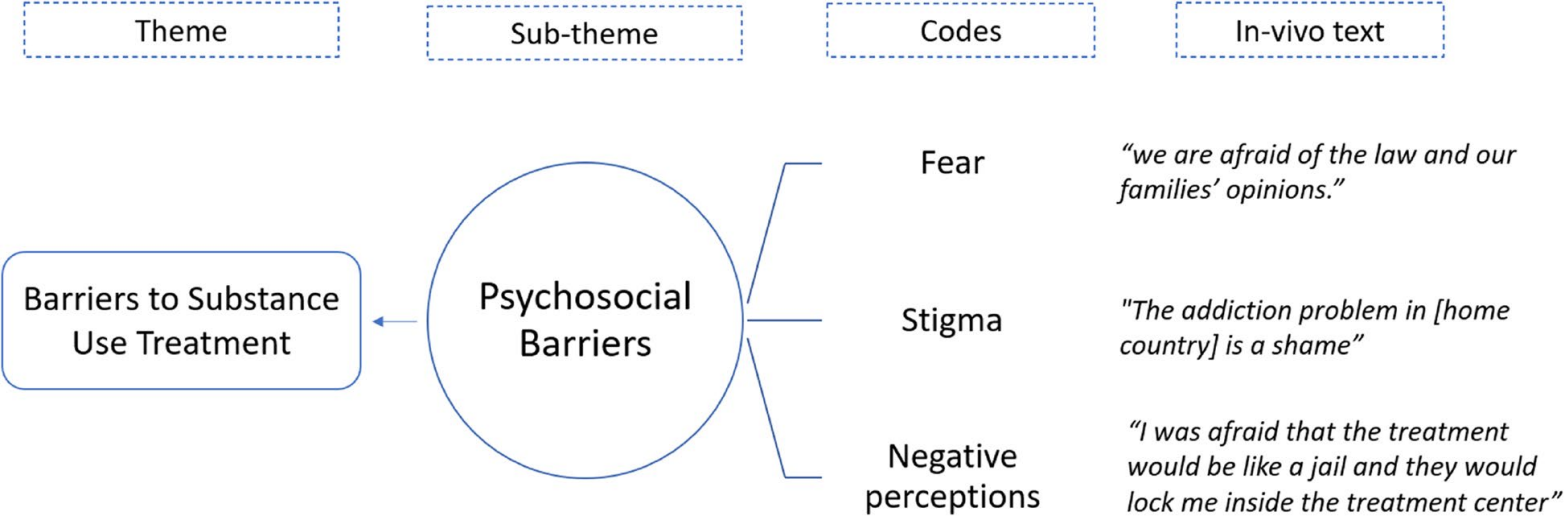
An example of thematic analysis from an in-vivo text to a theme

The interviews were anonymized, transcribed by the interviewer, and translated from Arabic to English by the interviewer, the translation was checked and reviewed by a specialized language service. Coding and categorization was facilitated by the software program MAXQDA 2022 (VERBI Software, 2021). The thematic analysis was conducted based on Braun and Clarke [36]. E.A.S and F.K. (native Arabic and German researchers, respectively) independently reviewed and initiated coding using an iterative approach. We drew the initial codes and provided a case summary after reviewing all transcripts from two rounds. By combining the inductive and deductive approaches, an initial set of conceptual codes was drawn, each code was defined, and a codebook was checked to determine whether the code was appropriately assigned. The coded data were then categorized and linked into categories by relationships. Links between these categories were established, from which sub-themes and main themes emerged. Further details are illustrated in Figs. 1 and 2. Additionally, a descriptive analysis (*Mean* [*M*] and *Standard Deviations* [*SD*]) was conducted for the participants’ demographic data and the ISI scores, as illustrated in Tables 1 and 2, respectively.

**Table 1:** Characteristics and demographics of thirteen professionals working in social and health institutions in Berlin

I.D.	Gender	Age	Country of origin	Job	Work Experience
Prof.1	F	31	Syria	Social worker	1–5 years
Prof.2	M	66	German	Social worker	> 25 years
Prof.3	F	39	German	Social worker	11–15 years
Prof.4	F	28	German	Social worker	1–5 years
Prof.5	F	34	German	Social worker	6–10 years
Prof.6	M	36	German	Social worker	1–5 years
Prof.7	F	32	Afghanistan	Nurse	1–5 years
Prof.8	F	49	German	Service provider/Social worker	11–15 years
Prof.9	F	28	German	Social worker	1–5 years
Prof.10	F	27	Jordan	Service provider/ Social worker	1–5 years
Prof.11	F	22	Syria	Service provider/ Social worker	< 1 year
Prof.12	F	39	Syria	Social worker	6–10 years
Prof.13	M	55	German	Social worker	1–5 years

**Table 2:** Demographic data and Insomnia Severity Index as reported by thirteen Arabic-speaking refugees receiving treatment for substance use in two rehabilitation centers in Berlin

ID-treatment setting	Age & Gender	Marital status	Country of origin	Deutsch	Education	ISI	Insomnia severity
SU1-r	52/m	Divorced	Tunisia	Intermediate	Secondary	19	15–21 moderate /clinical
SU2-r	22/m	Single	Somalia	Beginner	Secondary	1	0–7 not clinical
SU3-r	32/m	Single	Lebanon	Intermediate	Primary	26	22–28 severe/clinical
SU4-r	33/m	Single	Egypt	Beginner	Secondary	11	8–14 sub-threshold
SU5-nr	48/m	Single	Syria	Beginner	Secondary	22	22–28 severe/clinical
SU6-nr	47/m	Married	Lebanon	Beginner	Secondary	13	8–14 sub-threshold
SU7-nr	31/m	In relationship	Lebanon	Intermediate	Primary	18	15–21 moderate /clinical
SU8-nr	24/m	Single	Lebanon	Intermediate	Primary	8	8–14 sub-threshold
SU9-nr	21/m	Single	Egypt	Beginner	Secondary	14	8–14 sub-threshold
SU10-nr	27/m	Married	Yemen	Beginner	Secondary	14	8–14 sub-threshold
SU11-nr	43/m	Single	Libya	Beginner	Primary	19	15–21 moderate /clinical
SU12-r	37/m	Single	Syria	Intermediate	Primary	10	8–14 sub-threshold
SU13-r	30/m	Single	Iraq	Fluent	Secondary	12	8–14 sub-threshold

*SU (substance use) * r (residential) *nr (non-residential) *ISI (Insomnia Severity Index)

#### Cultural and linguistic background of the researcher

To minimize the challenges in interviewing distressed populations about sensitive topics, building trust and sharing a common cultural and linguistic background are considered effective facilitators in qualitative approaches [37, 38]. The main author (EAS) comes from a similar cultural background; she is a pharmacist who conducted similar studies among Arabic populations in clinical and social settings in two countries (Yemen and Jordan) [39–41]. She is familiar with the culturally diverse refugee and SUD contexts because of her country of origin (Yemen), which hosts diverse refugee groups (Somalian, Ethiopian, and Syrian). In terms of the language, although Arabic is a common language among all Arabic countries, each Arabic region has groups of dialects on its own [42]. The researcher spent several years studying and working in three Arabic countries, which allows her to speak and understand different dialects. Respondents are native Arabic speakers as well, originally coming from eight Arabic countries.

## Results

### Participants

Two groups of participants were recruited: 13 professionals and 13 refugees. The professional group ranges in age from 22 to 66 years (*M* = 35.9, *SD* = 11.8), with an average of five to nine years of work experience (Table 1).

The refugees ranged in age from 21 to 52 years *(M* = 35, *SD* = 11.9), their average time in Germany was 6.3 years, and they originally came from eight Arabic countries (Table 2).

Only one participant had no clinically significant insomnia. In contrast, seven had sub-threshold insomnia scoring 8–14, three had moderate insomnia scoring 15–21, and two had severe insomnia scoring 22–28 in the *ISI* (Table 2). The ISI’s Mean *was* 14.3 with standard deviation 6.5.

### Thematic results

**Fig. 3:**
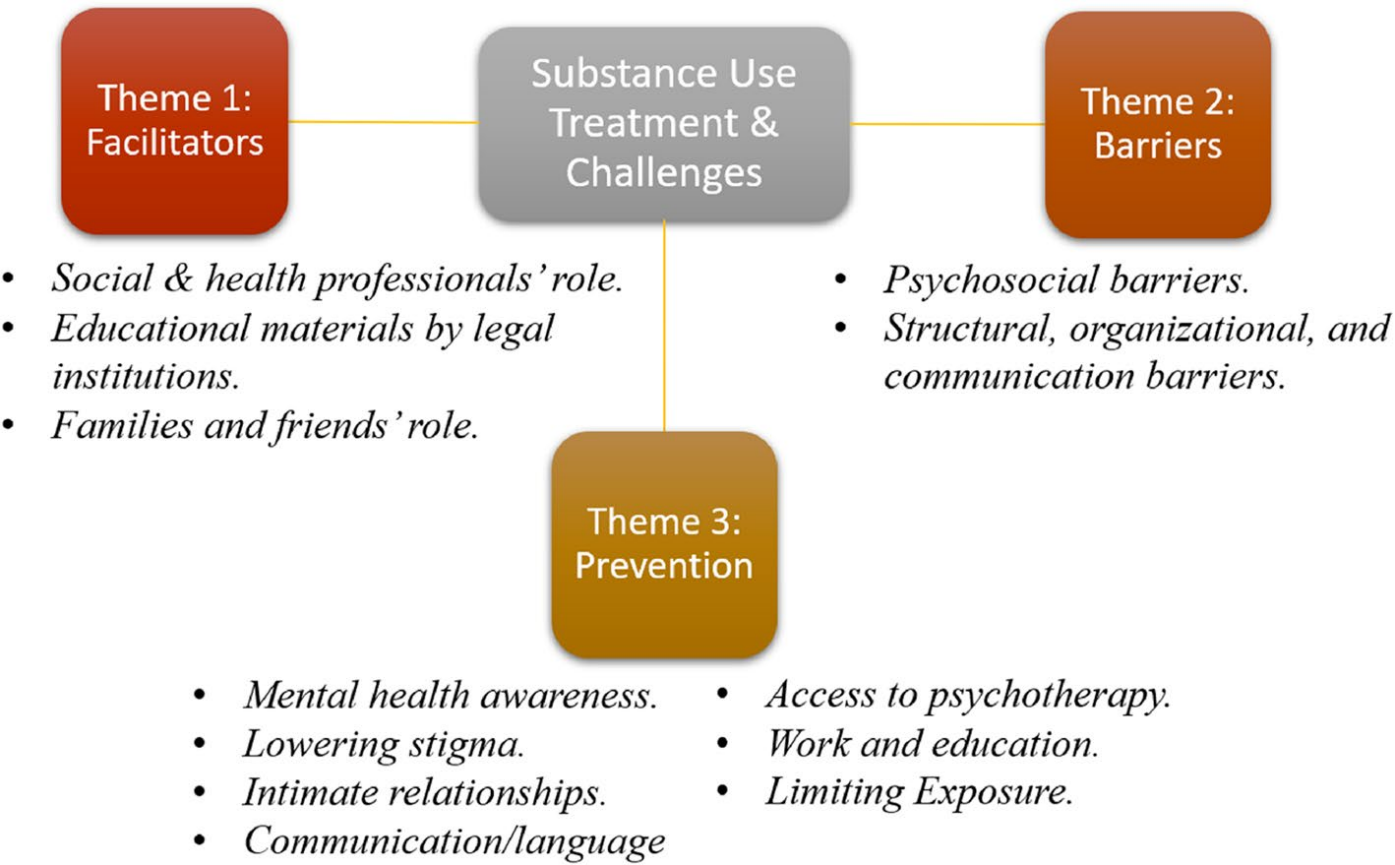
Thematic findings as resulted from the content analysis of 26 interviews in this study

Results were categorized in three themes that relate to the research questions, as illustrated in Fig. 3.

### Theme 1: the treatment is facilitated by institutional and emotional support

Both groups in this study (professionals and refugees) explained the value of guidance and emotional support as facilitating factors in seeking help for SUD treatment. The main supportive resources were professionals, legal institutions, and emotional support by family members and friends.

#### Referral by social and health professionals

Refugees with SU contacted professionals, as reported by the majority of professionals. This is consistent with most refugees’ responses, where they explained that professional contact and guidance facilitated their treatment.

"Some clients [the refugee] come alone, and others come through a social environment or through a practice or a counseling center." (Prof.1)

"I decided to seek help and talk to the social worker in the refugee camp, and she helped me and guided me to this treatment." (Refug.4)

As explained by refugees, their decision to seek treatment was in a health institution after having a near death experience due to overdose and toxicity, for example, a heart attack, where the physicians and healthcare providers who saved their lives warned the participants about future consequences.

"I collapsed on the street, and the ambulance took me to the hospital; the doctor said I was about to die. I knew that I must stop drinking alcohol, and I determined to attend the treatment." (Refug.2)

"I have been rescued by the ambulance twice. My heart was about to stop because of Coke [cocaine] overdose. The doctor said that my heart will stop beating because of this large dose" (Refug.3)

#### Educational materials by legal institutions

The role of legal institutions, such as prisons, was mentioned as a treatment facilitator, where educational material about SU was provided. This information was reported by the minority of refugees in the sample who had legal penalties and spent an average of 12–18 months in prison in Germany. In addition, the referral by legal institutions was mentioned only by the refugee sample.

"It had happened when I was in jail. After watching a movie about crystal [methamphetamine] and how it destroys the lives of young girls and boys, I panicked. I was scared about my health; then, I realized that I must seek treatment as soon as possible."^3^ (Refug.1)

#### Supportive role of families and friends

Almost half of the professionals in this study mentioned that family members or friends of refugees also facilitated connections with the treatment. The minority of refugees highlighted the helpful roles their families and friends played in encouraging them to seek help.

"I know about the problem of substance use by the refugee him/herself, or his family and friends." (Prof.4)

‘My wife supported and encouraged me to seek treatment” (Refug.1)

‘My friends worried and carried about me, advising many times to seek treatment’ (Refug.12)

### Theme 2: the affected refugees struggle with complex contextual barriers to access SUD treatment

#### Psychosocial barriers

Consistent with the majority of the professional group, the refugee group reported complex psychosocial barriers in terms of stigma, negative perception, and fear of substance use treatment. Almost all refugees in this study mentioned stigma as a barrier that could be higher in their home countries, concomitant with a fear of legal penalties. Most professionals stated that stigma also exists in the host country, which limits access to effective treatment.

"In my view, they are stigmatized and marginalized just like all other drug addicts. The degree of stigmatization and exclusion could even increase, depending on where they live and their social milieu." (Prof.4) "Because of their self-image, they are hesitant to seek help or may interrupt their treatment." (Prof.8)

"The addiction problem in [home country] is considered a shame, the first thing, they will see you as a sick person and then go to jail with a high criminal penalty; we are afraid of the law and our families’ opinions.” (Refug.1)

Although most refugees in this study did not clearly state their family’s attitude regarding their substance-use problem, the minority indicated that they sought treatment before their families in their home countries knew about their SU.

“One of my friends said that he would record me on a video during this paranoia he said that I was doing weird things and, in the end, collapsed, crying and shouting. He said he would send this video record to my family back there in [home country] to convince me to seek treatment." (Refug.1)

The majority of professionals experienced difficulties with the families of the refugees in terms of denying or hiding the problem, feeling ashamed, or isolating the person who used substances from the family.

"This is the most important part, family and friends should be the support and encouraging source, but unfortunately, they usually hide and may exclude the user from their lives." (Prof.8)

Negative perceptions of SUD treatment and fear are common barriers to treatment. Similarly, the majority of the refugee group emphasized their fear of SUD treatment because of the false perception that it might be like a prison, similar to their home countries.

"In many Arab countries there are no addiction counseling centers. You have to do outreach work in emergency shelters. You have to explain how the help system works in Germany." (Prof.1)

"Fear is another barrier, and they are afraid of the treatment because they do not understand the system of addiction treatment here." (Prof.10)

"When my wife suggested going to the addiction treatment, I was afraid that the treatment would be like a jail and they would lock me inside the treatment center, you know like the treatment in our countries, so I refused and went to the Mosque; praying might be the best medicine." (Refug.1)

#### Structural, organizational, and communication barriers

The majority of professionals mentioned several challenges in accessing mental health services including language challenges, long waiting times, and limited health insurance as the most common structural and organizational barriers to refugee SUD treatment.

"Long waiting times, insurance partly not available, language barriers in the therapy and accessing the therapy is mostly not possible without social service or others." (Prof.9)

Although the refugees did not mention the organizational and structural barriers as precisely as the professionals did, the majority stated the challenges in understanding physicians and their need to talk with professionals about their current mental problems, such as trauma flashbacks and insomnia, which aligns with the self-reported insomnia severity index that was illustrated previously in Table 2.

"I need to talk to a specialized physician to tell him everything I feel. I am struggling with sleep issues now, and the same shocking memories of my brother's death keep coming back" (Refug.1)

"I tried psychotherapy, but it did not work. I could not understand them." (Refug.2)

"I do not understand German; sometimes, I don't know what the doctor says." (Refug.9)

### Theme 3: individual and community preventive strategies

#### Preventive strategies on the individual level – “refugees”

Both groups consistently explained the value of mental health awareness and relevant information as preventive strategies.

#### Mental health awareness

Almost all professionals in this study emphasized the value of improving mental health awareness by reaching out to refugees and their families as a preventive strategy and facilitator of treatment.

"Familiarize refugees with the treatment system and its different types, and treatment doesn't mean punishment or a future barrier to finding a job." (Prof.10)

Professionals recommended promoting resilience as a preventive strategy. The refugee group similarly explained how the information they received in prison and/or treatment centers helped them better understand themselves and manage their negative emotions.

"By fostering their own resilience and trying not to use substances to cope with their emotions and stresses." (Prof.4)

"Wow, I liked the group discussion here very much. I learned more about myself. I can understand myself and see my mind from the inside. I knew that I needed to manage my life and my feelings so I would not be overwhelmed with stress or any bad feelings that seduce me to consume illicit drugs again." (Refug.7)

#### Lowering stigma

The majority of professionals emphasized the significance of lowering stigma among refugees and their families as a treatment facilitator and a preventive approach. Similarly, most refugees explained the difference in their perceptions regarding SU and treatment, in which SU in the host country is considered just a disease that needs healthcare responses while the high-level stigma in their home countries might hinder treatment.

"To provide lectures about substance use and to lower the stigma among families and the refugees' community in general." (Prof.8)

"In my community, if I am drinking alcohol, everyone will hate me, but here, they know that it is a disease and helped me to recover." (Refug.2)

#### Intimate relationships

While the professional group did not mention it, “intimate relationships” were mentioned as a preventive strategy by the majority of refugees. The “intimate relationships” mentioned included improving long stable relationships, making a family, or caring for their existing families.

"Maybe if I stayed alone like this without a partner or family, I would relapse." (Refug.5)

"I need to get back to my family, my daughters, they talked to my daughters about my problem, and now they visit me and encourage me. It was the first time I invited them to a restaurant two weeks ago; I was so happy." (Refug.1)

#### Community structure and organization level – “host community”

Both groups (refugees and professionals) identified three prevention strategies based on further improvements at the organizational and structural levels in the host society: “work and education,” “assistance in communication/language,” and “limiting exposure to substances.” Furthermore, the majority of professionals considered “facilitating access to psychotherapy” as a preventive strategy that could be achieved by working on financial and language issues to cover costs and providing more translators. Access to psychotherapy was mentioned by most of the professionals in this study emphasizing on the need for more translators, psychiatrists, and the need to provide psychotherapy insurance for refugees.

"Providing more translators, more initiative psychotherapists, and health insurance for refugees should cover the psychotherapy costs." (Prof.11)

The majority of professionals and refugees emphasized the importance of language and communication as a significant strategy to facilitate counseling and substance use treatment. Some refugees in our study were interested in attending group discussions as a communication and learning opportunity from others’ experiences.

"It is necessary to open up existing services for people without German language skills in the sense of intercultural openness, and not to make counseling and treatment dependent on German language skills." (Prof.4)

"I need to understand the language and communicate with people." (Refug.5). "I would like to participate in group discussions with others and know others' experiences." (Refug.7)

The majority of professionals and refugees in this study mentioned that providing job and training opportunities were considered as a preventive strategy.

"Provide job opportunities, so they don't have empty time to waste." (Prof.8)

"Ausbildung [vocational training or apprenticeship] is all I need, a chance to find a job, at least to guarantee that I will not spend more time in the streets again." (Refug.3)

The majority of refugees perceived the limitation on exposure to substances as a preventive strategy, but a minority of professionals mentioned a similar preventive strategy. Limiting the exposure might be achieved by avoiding certain places and contacts as perceived by the professionals. Refugees reported their approach in avoiding similar places and relevant contacts as much as they could.

"If possible, do not intensify contact with people who use drugs, and avoid places known for drugs such as certain parks and subway stations." (Prof.12)

"It is an endless battle with myself when I see these people using drugs around me in the stations, coffee shops, and under bridge. I don't want to see people consume drugs in public." (Refug.5)

## Discussion

This study explored the perspectives and experiences of professionals and Arabic-speaking refugees regarding the facilitators and challenges of effectively accessing substance-use treatment services in Germany. The three main findings of this study advance our knowledge of SUD treatment among refugees [43, 44].

First, emotional support and informative guidance effectively connected the refugees to SUD treatment. After being rescued from an overdose complication, refugees were guided through direct contact with counseling or health service professionals. Legal authorities, such as prisons, can act as resources when they effectively inform inmates about potential treatment options. Educational materials about SU in a refugee camp setting might also be a helpful resource. Notably, the “refugee camp setting” was not mentioned as a resource for similar educational materials on SU. Relevant findings on implementing educational guidance for SU in refugee camp settings remain scant, particularly among Arabic-speaking refugees resettled in Western nations, who seem to have lower help-seeking behaviors and poor mental health awareness [45, 46]. Findings from recent studies conducted among Arabic-speaking refugees [47, 48] are in line with our findings emphasizing the value of providing cultural and linguistic educational materials about SU and access to treatment in Germany, which will help promote help-seeking behaviors and mental health awareness [49].

The second key element of this research was the complexity of psychosocial and communication barriers to accessing SUD treatment. Stigma and fear of treatment were the main psychosocial barriers, which is consistent with the literature [6]. Besides the fear of reporting SU due to their sensitive legal status in host countries [50, 51], refugees with SU struggle with multiple sources of stigmatization from the host community and their communities [6]. Mental health problems associated with stigma and discrimination among Arabic-speaking refugees in Germany have also been reported [52–54]. Consequently, stigma and fear of treatment complicate refugees’ mental health and may worsen their condition [6, 55]. Refugees from the Arab region and low-middle income countries might be unaware of the SUD treatment process and fear the legal consequences of reporting their SU [56, 57]. To improve awareness and resolve misconceptions about the treatment process, providing educational and informative materials to Arabic-speaking refugees could help minimize similar psychosocial barriers [47, 57].

To ensure effective communication to refugees about SU, it is essential to consider the “language burden” which was mentioned during the different levels of the treatment journey, accessing the treatment and therapy sessions. The refugees reported language challenges while talking to specialists about their mental problems during treatment (e.g., flashbacks, insomnia, and paranoia). Literature has reported that language barriers restrict effective communication with professionals during psychotherapy sessions [58, 59], and similar mental problems can lead to a higher chance of relapse [60, 61].

Developing linguistic and cultural treatment models has significantly increased refugee participation in treatment programs [51, 62]. In Germany, there is low-level implementation of cross-cultural approaches to psychosocial services due to challenges in financing language mediation in mental health services [59, 63]. Recent studies recommended adapting culturally sensitive approaches among refugees who use substances in Germany [49, 64]. Nevertheless, further studies are required to determine the acceptability and effectiveness of these approaches [64]. It is also essential to enhance the integration of mental health services with the host community, which could be achieved by implementing multicultural treatment models for non-German speakers as part of German Treatment services.

Third, individual and community preventive strategies should be implemented. Individuals’ mental health awareness is a cornerstone of accessing SU treatment, considering that mental health concepts are perceived differently across cultures [65]. The diversity in perceptions was confirmed in our findings. The refugee group mentioned “intimate relationships,” more specifically “family” as a preventive strategy for SU while professionals mentioned “access to psychotherapy.” This result emphasizes the different perspectives and supportive resources of mental health across cultures. In addition to faith, family is the second most common cornerstone of Arabic communities and is considered a primary source of emotional support [66, 67]. Some cultural groups prioritize spiritual and intimate relationships as supportive and preventive strategies while psychotherapy is stigmatized. We recommend that more efforts are needed among refugee families and religious institutions to enhance awareness regarding the host country’s psychotherapy system and mental health services. It is worth mentioning that transcultural psychiatry can also play a significant role in this context, where the professionals would be familiarized with the diversity of supportive systems based on the refugees’ cultures and beliefs [68, 69].

Consequently, it is essential to consider diversity in cultural values when tailoring mental health awareness programs among Arabic-speaking refugees resettled in Western communities [12, 24]. It might also help to lower stigma if families engage with affected refugees in similar culturally tailored programs [49]. Another preventive strategy mentioned by participants is “work and/or occupational training,” which aligns with relevant studies conducted in Germany that showed that an apprenticeship or regular occupation was associated with lower psychological distress among refugees [70]. In line with the World Health Organization’s demand [71], decision makers should facilitate access to the labor market as a preventive intervention to promote refugees’ mental health [5].

Finally, the professionals in this study recommended facilitating “access to psychotherapy” for refugees as an intervention. The treatment gap in psychotherapy for refugees in Germany has been discussed previously [48, 72]. Due to the legal status of refugees, which limits access to the health system, there is a lack of trained therapists and psychotherapeutic treatments for culturally diverse groups [48, 73]. We believe that it is crucial to facilitate access to effective psychotherapy and integrate mental health into primary healthcare for refugees in Germany [74] for the screening and early detection of psychiatric disorders to minimize the risk of SU and relapse [75].

## Limitations

This study has a few limitations and proposes several implications for future research and practice with Arabic-speaking refugees. First, we used a convenience sample of participants who receive treatment in rehabilitation centers. Therefore, it is likely that our study participants and the perspectives they shared are not representative of the broader Arabic-speaking refugees in Germany. Second, we initially recruited males and females who use substances, yet we successfully interviewed males only while females refused to participate. Women who use substances face addition barriers to participate in similar studies, which might limit the gender diversity of the sample and induce a potential risk of sampling bias. Although the interviewer has a similar sociocultural background to the participants, she was careful in using gender and religion-neutral language. For future research, it is significant to pay attention to the interviewer’s appearance, gender, and age that might affect the interview and cause potential bias.

Finally, the purpose of this study’s insomnia severity index (ISI) was to identify insomnia among refugees receiving treatment for substance use. In alignment with the data extracted from the interview, the ISI data in our research is valuable in emphasizing that refugees struggle with insomnia during their recovery and complain of poor communication with specialists in this regard. However, it is significant to note that incorporating substance use measures (e.g., substance use patterns, severity, etc.) alongside the ISI enhances understanding of the complex interplay between substance use and insomnia in future research, leading to more effective prevention, intervention, and treatment strategies.

## Conclusion

To our knowledge this is the first in-depth study to explore the treatment experiences of Arabic-speaking refugees and professionals in Germany regarding SU. Findings highlighted the psychosocial and structural challenges of connecting the affected refugees with treatment services in the host community. Our findings emphasize the sensitivity and cultural variations of perceptions regarding mental health and the treatment of SU. In addition to improving more preventive strategies, further in-depth studies are needed to understand the acceptance and effectiveness of SU treatment programs among different refugee groups.

### Supplementary Information

**Additional file 1.**

**Additional file 2.**

**Additional file 3.**

## Data Availability

The data supporting the conclusions of this article are included in the manuscript. In addition to Interview guidelines (Supplementary files 1 & 2).

## Abbreviations

SU: Substance Use
SUDs: Substance Use Disorders
UNHCR: the United Nations High Commissioner for Refugees
PTSD: Post Traumatic Stress Disorder
ISI: Insomnia Severity Index
CETA: Common Elements Treatment Approach

## Footnotes

1 Street drugs such as opiates, cocaine, ecstasy, marijuana etc.

2 Refugee sample recruited from two rehabilitation centers (a) Drogennotdienst GUIDANCE, (b) ADV, Suchthilfe NOKTA, Berlin, Germany.

3 This educational material in prison was perceived helpful from the individual’s perspective and personal experience level, yet it should not be generalized as an approach for substance use counseling.
