# Supplementary material for: Challenges in substance use treatment as perceived by professionals and Arabic-speaking refugees in Germany
Source: Subst Abuse Treat Prev Policy. 2023 Nov 17;18:69. doi: 10.1186/s13011-023-00576-5 (PMC10656940; doi:10.1186/s13011-023-00576-5)
Supplement: Supplementary file 1 — Additional file 1. [file 13011_2023_576_MOESM1_ESM.pdf]

Supplementary Files (1) The Interview Guide with Refugees and Professionals

Interview with Arabic-speaking Refugees in rehabilitation Centers in Berlin

Face -to-Face Interview

**Main questions**	***Prompts and Probes***
1. Would you like to talk briefly about yourself?	Probe<br>*Would you elaborate on…..*
2. When did you realize you have a substance use problem and decide to seek help?	Prompts<br>*Why did you feel it was time to seek help?*
3. Who was the first contact you talked with about your problem?	Probe<br>*Would you explain further on how….*
4. How was your experience with the treatment so far?	Prompts<br>- *Why did you find it difficult? OR*<br>- *Why did you find it good/ beneficial?*<br>- *How do you feel now*
5. Do you have any information about the side effects of these substances?	Prompts<br>*Who provided this information?*
6. What further information do you need to know about substances and addiction? If there is any.	
7. What do you think the reason(s) might make you relapse again when you finish your treatment?	Probe<br>*Would you elaborate on…..*
8. In your opinion, what might you need in order to overcome the addiction completely?	Probe<br>*Would you provide more examples…*

Online Interview Guide with Professionals

Via SoSci-Survey Platform

1. As a service provider, how do you typically hear about the problem of drug abuse among some Arabic-speaking refugees?

   By the refugee him/herself
   By one of the refugee's family or friend
   Others

   ---

2. What is your perception: How do Arabic-speaking refugees who abuse drugs, are seen by the general public?
3. To what degree does this influence your work/prevention?
4. What is your perception: How do Arabic-speaking refugees who abuse drugs think or feel about their own behavior?
5. How does this influence your work/prevention?
6. What is your perception: How do you think their close family and friends think about their behavior?
7. How does this influence your work/prevention?
8. What is your perception: How do you think other Arabic-speaking refugees think about such behavior?
9. How does this influence your work/prevention?
10. . How do refugees react to the advice that they have to attend treatment for substance abuse?
11. What are the barriers to accessing drug abuse treatment for Arabic refugees?
12. What are the facilitators for accessing drug abuse treatment for Arabic refugees?
13. How can service providers/professionals help in the treatment/ prevention of drug abuse among Arabic-speaking refugees?
14. How can Arabic-speaking refugees avoid/ prevent the drug abuse problem?
15. What can others (Family, friends, society) do to participate effectively in the treatment/ prevention of drug abuse among Arabic-speaking refugees?

16. Do you think that Arabic-speaking refugees need more workshops (preferably in Arabic language) to focus on the drug abuse problem? If yes, please provide us with more detailed titles/topics.

    Yes
    No

    Yes, such as
