# Supplementary material for: Challenges in substance use treatment as perceived by professionals and Arabic-speaking refugees in Germany
Source: Subst Abuse Treat Prev Policy. 2023 Nov 17;18:69. doi: 10.1186/s13011-023-00576-5 (PMC10656940; doi:10.1186/s13011-023-00576-5)
Supplement: Supplementary file 3 — Additional file 3. [file 13011_2023_576_MOESM3_ESM.pdf]

Appendix I “Consent Form”

Informed Consent Form

Data of participants in the study

Last name:	First name:
Place of birth:	Date of Birth:
Tel.no.:	Street:
Zip. Code:	City:

This is an acknowledgment of my willingness to participate in the research entitled “Substance abuse: In-depth interviews with refugees in addiction and rehabilitation centers in Berlin”.

- I acknowledge that the researcher “*Ebtesam A. Saleh*” has informed me about the nature of the research project and its objectives clearly and completely. I certify that I have read and understood the above "Participants’ Information Sheet" and the text of this “Informed Consent Form”. The researcher has answered all my inquiries regarding recording and archiving of data.
- I had enough time to ask questions and take decisions.
- I have been notified that I can withdraw my consent whenever I want to, and then any of my data will be deleted.
- I received a copy of the participant's information form and consent. The original copy will remain with the researcher.
- I declare that I agree to register my data / personal and health information for the study participant and to use it within the research project without mentioning any personal names or addresses. For example, when publishing in scientific journals and conferences.

Participants’ Name:	Researchers’ Name:
Date: …. /……/…….	Date: …. /……/…….
Signature:	Signature:
